# Sitosterolemia—10 years observation in two sisters

**DOI:** 10.1002/jmd2.12038

**Published:** 2019-05-28

**Authors:** Lara Veit, Gabriella Allegri Machado, Céline Bürer, Oliver Speer, Johannes Häberle

**Affiliations:** ^1^ Division of Metabolism and Children's Research Center University Children's Hospital Zurich Zurich Switzerland; ^2^ Division of Haematology and Children's Research Center University Children's Hospital Zurich Zurich Switzerland; ^3^ Institut für Labormedizin Spital Thurgau AG Frauenfeld Switzerland

**Keywords:** *ABCG5* or the *ABCG8* gene, familial hypercholesterolemia, phytosterols, sitosterolemia, xanthoma

## Abstract

Familial hypercholesterolemia due to heterozygous low‐density lipoprotein‐receptor mutations is a common inborn errors of metabolism. Secondary hypercholesterolemia due to a defect in phytosterol metabolism is far less common and may escape diagnosis during the work‐up of patients with dyslipidemias. Here we report on two sisters with the rare, autosomal recessive condition, sitosterolemia. This disease is caused by mutations in a defective adenosine triphosphate‐binding cassette sterol excretion transporter, leading to highly elevated plant sterol concentrations in tissues and to a wide range of symptoms. After a delayed diagnosis, treatment with a diet low in plant lipids plus ezetimibe to block the absorption of sterols corrected most of the clinical and biochemical signs of the disease. We followed the two patients for over 10 years and report their initial presentation and long‐term response to treatment.

## 1 INTRODUCTION

Sitosterolemia (MIM #210250) is a rare, autosomal‐recessive disease characterized by accumulation of plant sterols in blood and tissues.^1^ Sitosterolemia was first described in 1973, when elevated plasma levels of plant sterols (sitosterol, campesterol, and stigmasterol) were detected in two sisters of European descent who had extensive tendon xanthomas but normal plasma cholesterol levels.^2^ Although the exact prevalence of sitosterolemia is not known, it may be underdiagnosed and in fact more common than the estimated incidence of 1 in 1 000 000.^3^, ^4^, ^5^ In fact, recent data indicate a prevalence of the disease more than 1 in ~200 000 individuals among the general population.^6^

**Figure 1:**
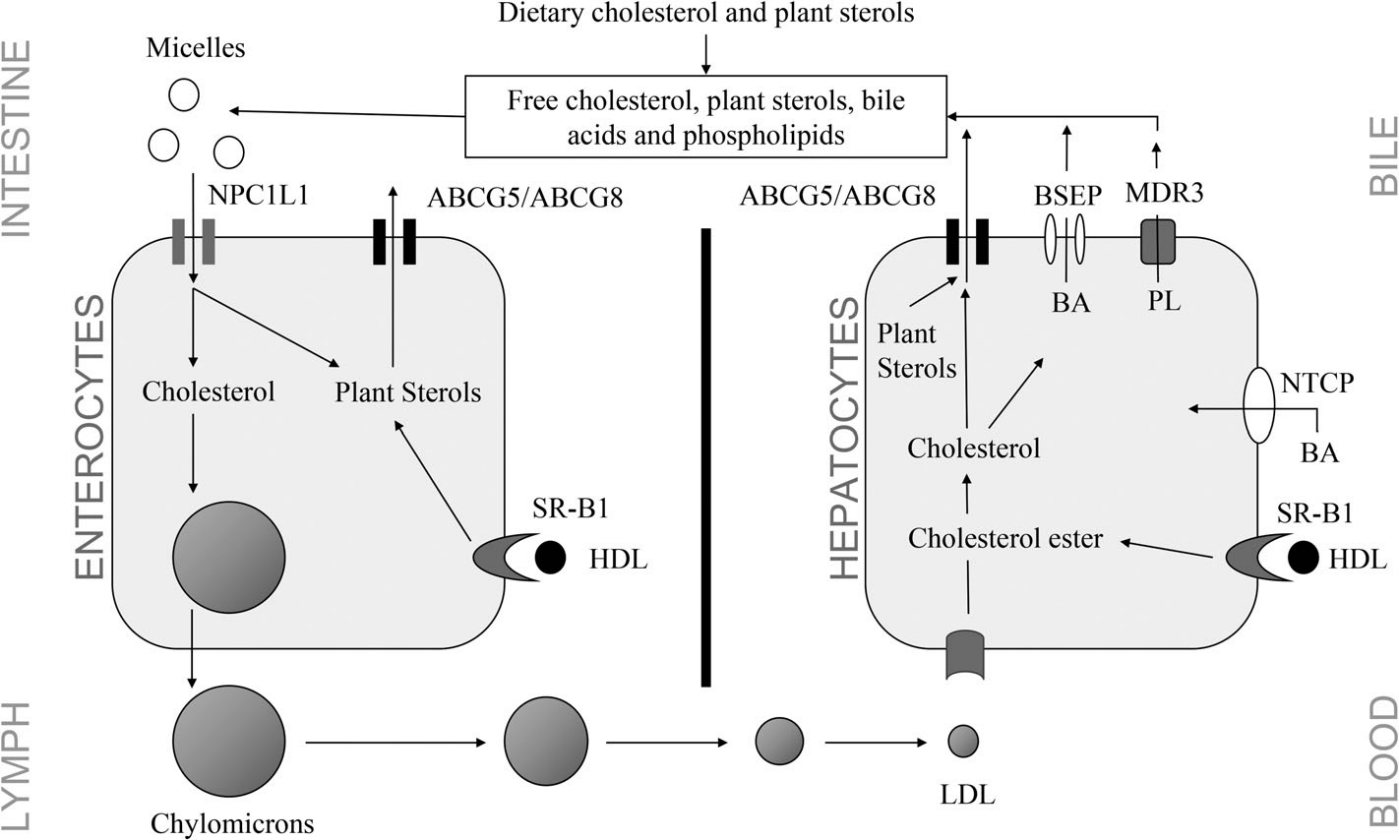
Model for absorption and secretion of cholesterol and plant sterols: Physiologically, the ABCG5/G8 transporter pumps absorbed nutrition sterols (absorbed through the Niemann‐Pick‐C1‐like 1, short NPC1L1) back into the intestinal lumen or into the bile, with a preference for non‐cholesterol sterols, if they are present. It occurs in the apical membrane of small intestine enterocytes and hepatocytes. BA, bile acids; PL, phospholipids; BSEP, bile salt export pump; BDR3, multiple drug resistance protein 3; NTCP, sodium/taurocholate co‐transporter; SR‐B1, scavenger‐receptor B1 (HDL‐receptor)

Sitosterolemia is caused by mutations in either the *ABCG5* or the *ABCG8* gene,^7^, ^8^, ^9^ two oppositely oriented genes located on chromosome 2p21.^10^ Inactivating mutations of both alleles at either the *ABCG5* or *ABCG8* locus cause sitosterolemia. A single report of a patient with sitosterolemia who is heterozygous for a mutation in both genes has been reported.^11^ While almost all Asian patients carry *ABCG5* mutations, Caucasian patients more often present *ABCG8* mutations.^1^, ^4^, ^12^, ^13^ *ABCG5* and *ABCG8* are coding for sterolin 1 and sterolin 2, respectively, which form an obligate heterodimer and ATP‐binding cassette transporter (Figure 1).^7^, ^8^, ^9^, ^13^ Loss of function of this transporter leads to increased absorption of all dietary sterols and thus to progressive accumulation of sterols. While plasma cholesterol is often normal in adult patients with sitosterolemia, pediatric patients can show severe hypercholesterolemia.^1^, ^5^

Clinically, sitosterolemia is very heterogeneous with a spectrum that extends from the patients being asymptomatic to early lethal cases.^5^, ^13^ Typical manifestations are listed in Table 1. The complete clinical expression of sitosterolemia may not be known due to underdiagnosis.^15^

**Table 1:** Possible clinical presentations of sitosterolemia

Arthralgia Tendon or tuberous xanthomas on the extensor surfaces Various hematological abnormalities: Mild anemia Stomatocytosis Hemolysis and increased osmotic fragility of the erythrocytes Macrothrombocytopenia Thrombocytosis Elevated transaminases Abnormal liver function tests Progressive liver disease Thickened heart valves Premature coronary heart disease Sudden death (age < 40 y) Endocrine insufficiency Only in children: severe hypercholesterolemia

*Note*: List of symptoms according to References 1, 5, and 14.

To diagnose this disorder requires the measurement of plant sterol concentrations in plasma using gas chromatography‐mass spectrometry (GC‐MS) or high pressure liquid chromatography. Standard enzymatic cholesterol analysis does not distinguish between plant sterols and cholesterol and therefore can lead to false high total cholesterol concentrations.^1^, ^5^ Mutation analysis can be made by sequencing the *ABCG5* and *ABCG8* gene.^5^, ^14^

Treatment of sitosterolemia aims to lower plasma plant sterols and, if elevated, cholesterol concentrations and to prevent complications.^5^, ^15^ To achieve this, a combination therapy is usually needed^5^: dietary restriction of both animal‐ and plant‐based sterols^15^, ^16^, ^17^; bile acid sequestrants, for example, cholestyramine^15^, ^16^, ^17^; and ezetimibe to reduce sterol uptake.^15^, ^18^, ^19^, ^20^, ^21^ Treatment results in a reduction in plasma concentrations of cholesterol and plant sterols and regression of existing xanthomas.^5^, ^15^ Thus, sitosterolemia is a treatable condition, especially when diagnosed and treated early, which underlines the importance of correct diagnosis and management of sitosterolemia.^5^

Here we report on two patients with sitosterolemia who were initially not recognized to have the disorder, who now have been treated for more than 10 years.

## 2 CASE REPORTS

Patient 1, first child of non‐consanguineous Bosnian parents, was referred to our unit at age 8.5 years after her parents gave a 1 year history of her developing bluish soft swellings on the extensor surfaces of both her knees and elbows. On physical examination, she was found to also have similar lesions on the buttocks and the Achilles tendons. Previously, one of the lesions had been biopsied and the diagnosis of “xanthoma disseminatum” was made. Her fasting blood total and low‐density lipoprotein (LDL)‐cholesterol levels were 12.1 and 10.2 mmol/L, respectively. Treatment with statins did not significantly lower her LDL‐cholesterol and the patient proceeded to develop xanthelasmas. Both of her parents had cholesterol levels that were only slightly elevated above the upper limit of normal, already raising suspicion about the possibility of a LDL‐receptor defect. Her younger sister was also tested and had a total cholesterol of 7.3 mmol/L and LDL‐cholesterol of 5.2 mmol/L. The family history beyond that was uneventful.

**Figure 2:**
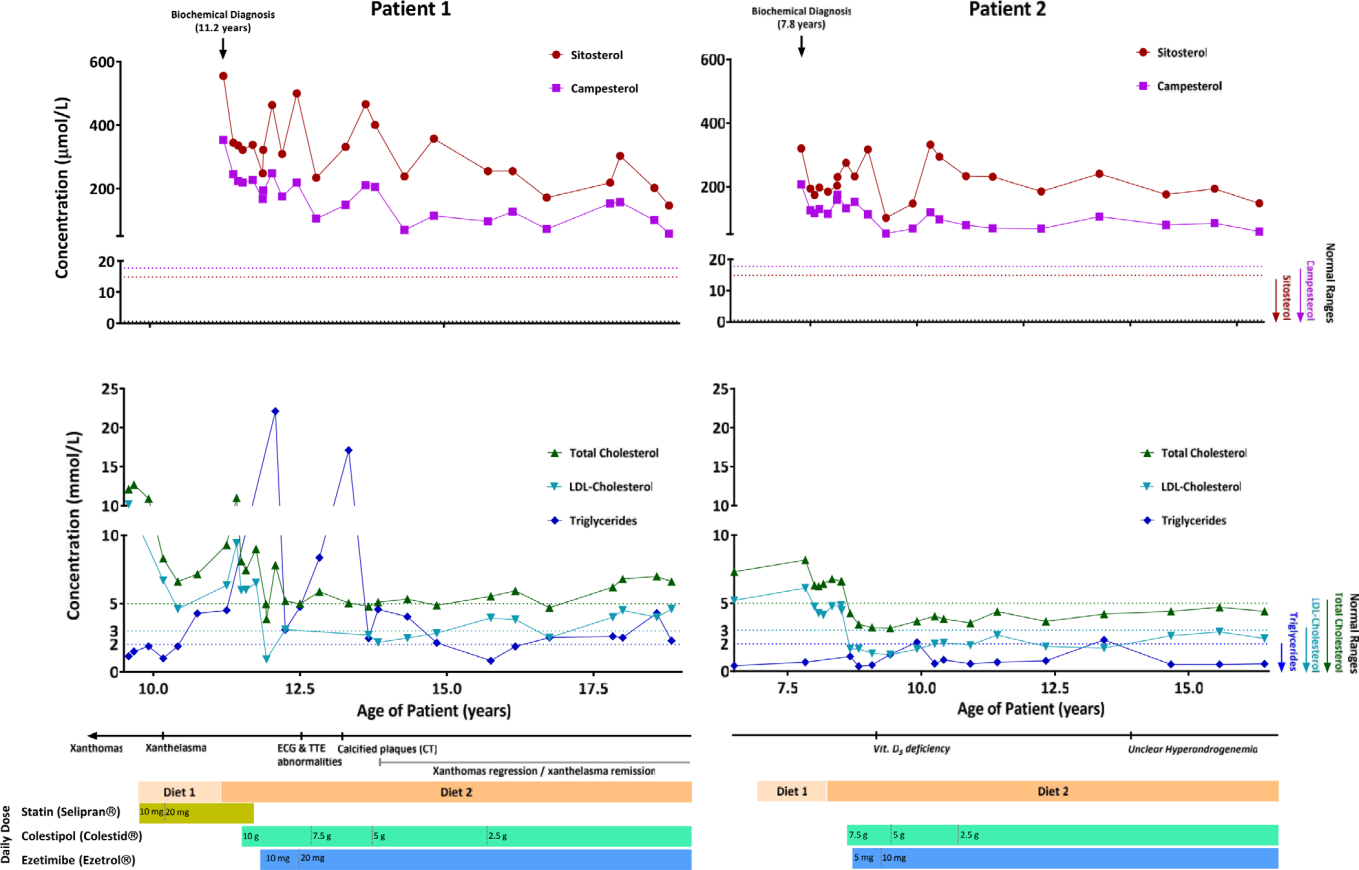
Lipid levels, symptoms, and treatments of both patients during the observation period

Sequencing of the coding exons of the genes encoding for the LDL‐receptor and apolipoprotein B in all family members did not reveal mutations. Finally, after 2.5 years, the plasma phytosterols were measured by GC‐MS and found to be very elevated (sitosterol 555 μmol/L, ref. 0.6‐14.88; campesterol 353 μmol/L, ref. 0.52‐17.65) (Figure 2). Genomic DNA from peripheral white blood cells was used for sequencing all exons of *ABCG5* and *ABCG8* (performed at University of Texas Southwestern Medical Center).^22^ This revealed that both patients were homozygous for a stop mutation at position 446 of *ABCG5* (p.Arg446*) and that both parents were carriers of the nonsense mutation.

When diet therapy for about 6 months plus colestid treatment for about 2 months did not lead to a reduction in plasma sterol levels, ezetimibe was added to the medical regimen. Treatment with ezetimibe led to a significant decrease of total and LDL‐cholesterol levels, as well as to a decrease in concentrations of phytosterols (Figure 2). In patient 1, treatment with ezetimibe led to an initial normalization of total and LDL‐cholesterol but levels increased later slightly above the reference ranges, while in patient 2 the concentrations of total and LDL‐cholesterol remained normalized until the end of the observation period (Table 2 and Figure 2). During treatment, xanthoma in patient 1 disappeared after several years with minimal bluish skin discoloration remaining in the respective areas of knees and elbows.

**Table 2:** Basic clinical and biochemical data of study patients

	Patient 1	Patient 2
Gender	Female	Female
Origin	Bosnia	Bosnia
Age at onset of symtoms	7.5 y	Showed no symptoms before diagnosis
Age at diagnosis	11 2/12 y (GC‐MS)	7 9/12 y (GC‐MS)
Mutation	Homozygous R446* on *ABCG5*	Homozygous R446* on *ABCG5*
Symptoms	Xanthomas (bilateral on knees, elbows, Achilles tendons, and buttocks). Xanthelasma on both lower eye lids.	No skin lesions
Complications	Echocardiography: Mitral‐ and tricuspidal valves at the neck hyperechogenic and thickened (12 10/12 y) ECG: disturbed repolarization in the chest leads. First degree AV block CT angiography: calcified plaques were found in the wall of the ascending aorta and subaortic in the left ventricular outflow tract	Stomach ache, vitamin D deficiency
Secondary diagnoses	Familial short stature	Epilepsy Hyperandrogenemia
Max. total cholesterol	12.7 mM	8.17 mM
Max. sitosterol	555 μM	332 μM
Max. campesterol	353 μM	207 μM
Mean of total cholesterol in the last 3 y	6.6 mM	4.5 mM
Mean of sitosterol in the last 3 y	217.2 μM	172.3 μM
Therapy	Diet low in plant sterols Colestid Ezetrol	Diet low in plant sterols Colestid Ezetrol

To investigate for known complications of sitosterolemia, platelet aggregation tests, thrombo‐FACS, platelet electron microscopy, ferritin, free hemoglobin (Hb), and Hb‐carbon‐monoxide (CO) were analyzed and found normal in both patients. Specifically, there was no evidence for the presence of hemolysis, abnormally shaped erythrocytes (stomatocytes) or thrombocytes (macrothrombocytopenia). Platelet aggregation was normal, as was expression of platelet surface proteins GPIb, IIb, and IIIa. All examined markers could be stimulated by thrombin.

**Figure 3:**
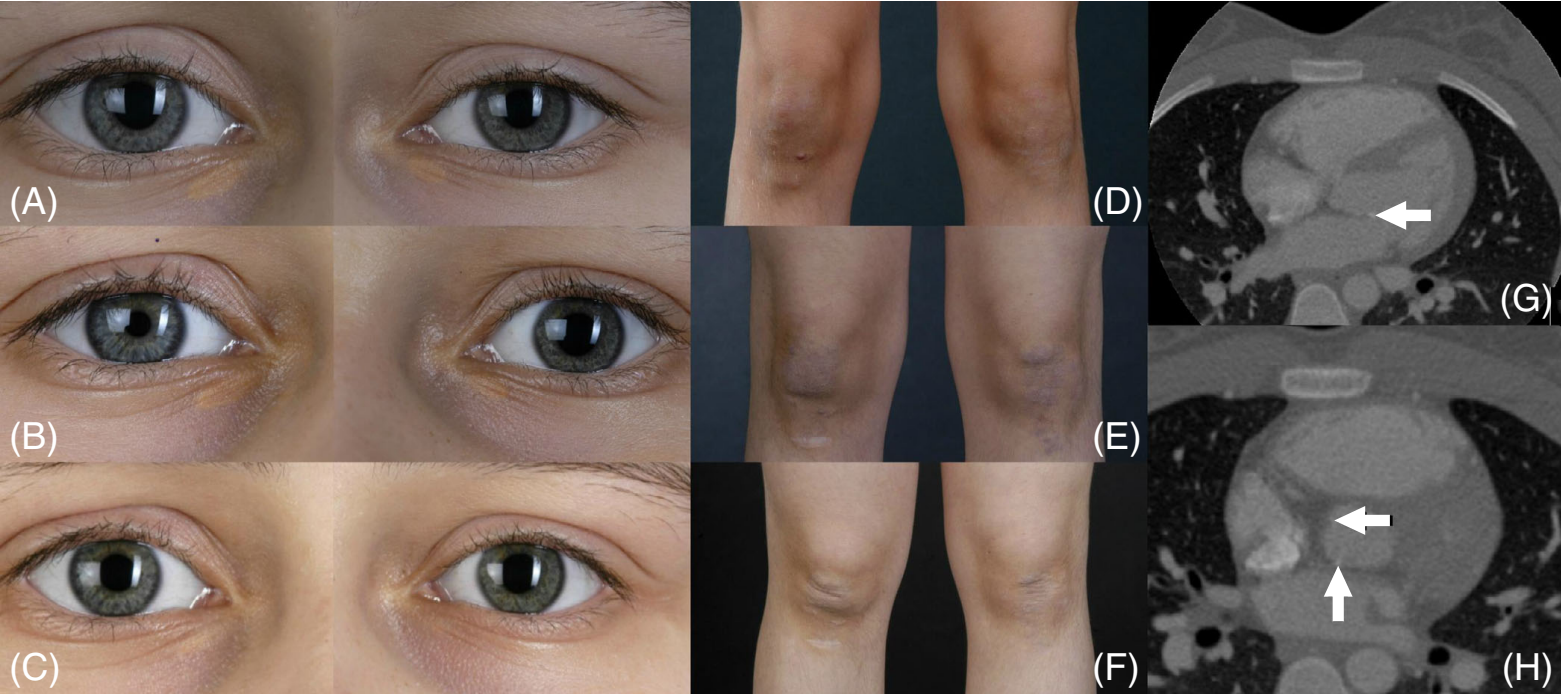
Clinical pictures and CT scan of patient 1. Xanthelasma at age 11.2 (A), 11.9 (B), and 13.3 years (C). Xanthomata at age 8.8 (D), 11.2 (E), and 13.3 years (F). CT scan with cardiac calcifications at age 13.5, arrows indicate site of calcifications (G, H). CT, computed tomography

In patient 1, echocardiography at 12.8 years of age showed hyperechogenic and thickened areas at the base of the mitral and tricuspid valves. One year later, a calcification had developed on the sinotubular junction, but without causing stenosis. These findings remained unchanged until the end of the observation period. When echocardiography showed signs of a disturbed repolarization, a CT angiography was done to exclude coronary artery involvement. This revealed normal coronary arteries but calcified plaques in the left ventricular outflow tract and in the wall of the ascending aorta (Figure 3). Patient 2 showed no cardiac involvement but additional morbidities including infantile epilepsy and mixed hyperandrogenemia (acne, mild hirsutism, and oligomenorrhoea).

Parents of the reported patients provided their consent to publication of this report.

## 3 DISCUSSION

Sitosterolemia is a rare and probably underdiagnosed autosomal recessive disorder of lipid metabolism. The clinical presentation is variable, making early diagnosis challenging. As the condition can lead to development of premature coronary atherosclerosis, which can be prevented by treatment, it is important that the disease be diagnosed early. Here we review the clinical presentation and successful management of two sisters with the disorder. In the index patient, the diagnosis of the disease was delayed by more than 2 years. After consultation with several specialities, the patient was assumed to have familial hypercholesterolemia (FH) based on elevated LDL‐cholesterol levels. However, treatment with statins alone failed, which is typical for sitosterolemia patients and a useful clue to the diagnosis. The initial misdiagnosis is likely the result of sitosterolemia being a rare disease, with less than 100 cases described to date.^23^ Besides several case reports, the largest case series reports 13 patients from 8 independent families, who were all treated at a single institution.^24^

Diagnosis of sitosterolemia requires a high level of awareness, as the standard lipid profiles provide no specific hints. Rather, specific phytosterol analysis must be requested.^23^ Another clue is that parents are normocholesterolemic, as it was in principle the case in the reported family with only slight cholesterol elevations in the parents. In case of absent hypercholesterolemia in the parents of a patient, and if xanthoma are present in typical body areas, physicians should include sitosterolemia in the differential diagnosis of elevated cholesterol concentrations. Confirmation of the disease may include analysis of *ABCG5* and *ABCG8* genes but mutation analysis is not required to make the diagnosis. Both our patients were homozygous for a previously reported stop mutation (c.1336C>T, p.Arg446*) in the *ABCG5* gene.^25^ This mutation was found in at least 19 patients from different origins: Iranian,^25^ Japanese,^12^, ^26^ Chinese,^14^ Romanian,^27^ Korean,^4^ Turkish,^28^ and Pakistani,^23^ plus further patients.^24^, ^29^, ^30^ Our patients are of Bosnian origin and therefore serve as examples for genetic heterogeneity at both loci. Previously, Asian patients more often carried *ABCG5* mutations, while Caucasian patients were more frequently affected by mutations in *ABCG8*.^1^, ^3^, ^7^, ^9^

The importance of early diagnosis and initiation of treatment is underlined by the different clinical and biochemical course in our patients: while patient 1 presented with multiple clinical signs including cardiac calcifications, patient 2 was asymptomatic. Also, maximum concentrations of cholesterol and of phytosterols in patient 1 were noticeably higher than in patient 2. While there are no apparent differences between patients with mutations in *ABCG5* or *ABCG8*,^8^, ^9^ there is considerable heterogeneity in the clinical and biochemical phenotypes of patients even with identical genotypes.^4^, ^13^, ^25^, ^31^ The cause of this variability is not known, but time of diagnosis and start of treatment may contribute. Additional factors may include *NPC1L1* polymorphisms leading to reduced sterol absorption and LDL‐cholesterol levels,^3^, ^31^, ^32^, ^33^, ^34^ variations in other lipid‐related genes influencing cholesterol absorption or other pathways,^34^ and differences in dietary sterol intake,^31^ the latter especially in infants consuming different diets.^15^

Management of our patients included cardiac investigations, regular monitoring of lipid and sterol profiles, and liver transaminases alanine aminotransferase and aspartate aminotransferase, alkaline phosphatase, and gamma glutamyl transferase to rule out liver involvement.^23^ Hematological investigations showed no stomatocytes, normal thrombocyte morphology and function, and no disturbance of hemostasis.^1^, ^5^, ^14^, ^24^, ^35^ Recently, it was suggested that “elevation of LDL‐cholesterol seems to be the major cause of development of atherosclerosis and not the elevation of sitosterols”^6^ rendering normalization of the cholesterol concentrations, as achieved in both patients, the primary treatment target.

Infantile epilepsy, present in patient 2, has been reported before in a single Chinese sitosterolemia patient,^31^ but a causal relationship is unclear. As well, sitosterolemia may have contributed to altered androgens and mixed hyperandrogenemia as plant sterols can serve as precursors for steroid hormones.^36^ However, the effect of phytosterols on the endocrine system is not completely understood. It is known that absorbed plant sterols mainly accumulate in the liver, gonads and adrenal glands indicating a high affinity to steroid‐synthesizing tissues.^37^ Further supporting a role of plant sterols, phytosterol‐enriched margarines led to a slight increase in testosterone in 10 women.^38^

In summary, elevated cholesterol concentrations are not always identical with FH, and sitosterolemia should be included as a treatable differential diagnosis. This is especially relevant if family screening does not confirm FH in at least one of the parents.

## TAKE‐HOME MESSAGE

Elevated cholesterol concentrations are not always identical with familial hypercholesterolemia, and sitosterolemia should be included as a treatable differential diagnosis. This is especially relevant if family screening does not confirm familial hypercholesterolemia in at least one of the parents.

## COMPLIANCE WITH ETHICS GUIDELINES

Lara Veit, Gabriella Allegri Machado, Céline Bürer, Oliver Speer, and Johannes Häberle declare that they have no conflict of interest.

This article does not contain any studies with human subjects performed by the authors. The family agreed to publication of this report.

## AUTHORS' CONTRIBUTION

L.V. and J.H. have planned the conception and design of the study. L.V., G.A. and C.B. have collected patients' data. O.S. has performed laboratory investigations. L.V. and J.H. have drafted the manuscript and designed the figures, which were revised by all authors.
